# Comparative Bibliometric Analysis of Herbal Medicine Research in Chinese and Iranian Complementary and Alternative Medicine (CAM) Clinical Trials: Short title: Iranian and Chinese CAM Trials

**DOI:** 10.31661/gmj.v15i.3573

**Published:** 2026-01-30

**Authors:** Shahram Shafa, Tayyebeh Zarei, Majid Vatankhah, Fatemeh Rahmanian, Ehsan Rahmanian, Sara Rahmanian, Mansour Deylami, Mojtaba Ghaedi, Amir Hossein Pourdavood, Lohrasb Taheri, Somayeh Mehrpour, Mehrdad Malekshoar, Bibi Mona Razavi, Fatemeh Eftekharian

**Affiliations:** ^1^ Research Center for Social Determinants of Health, Jahrom University of Medical Sciences, Jahrom, Iran; ^2^ Department of Anesthesiology, Critical Care and Pain Management Research Center, Hormozgan University of Medical Sciences, Bandar Abbas, Iran; ^3^ Department of Anesthesiology and Critical Care, Faculty of Medicine, Golestan University of Medical Sciences, Gorgan, Iran; ^4^ Department of Anesthesiology, Shahid Rajaee Heart Hospital, Tehran, Iran

**Keywords:** Bibliometrics, Herbal Medicine, Traditional Chinese Medicine, Persian Medicine

## Abstract

**Background:**

Traditional medicine, rooted in ancient history, resurged due to concerns over synthetic pharmaceuticals’ adverse effects. Persian medicine, emphasizing medicinal plants, aligns with global healthcare recommendations. Both Iranian and Chinese medicine have influenced medical knowledge historically. Patients worldwide value Traditional Chinese Medicine (TCM) for chronic and severe illnesses. This study goal was to conduct a bibliometric analysis of herbal medicine research in Iranian and Chinese CAM clinical trials to identify trends, patterns, and differences in research productivity and impact.

**Materials and Methods:**

This bibliometric study compared Chinese and Iranian CAM clinical trials using Web of Science data. It focused on RCTs, applying search terms for CAM and Chinese or Iranian CAM, with exclusion criteria to filter out irrelevant publications. No limitation was posed on publication time. The dataset included author information, article details, keywords, and citation history. It calculated the Annual Growth Rate (AGR) and used visual tools like the Three-Field Plot to illustrate associations. Additionally, Lotka’s Law was applied to author productivity, and a co-citation collaboration network was analyzed using Bibliometrix r package.

**Results:**

Key findings for Iranian CAM include 71 documents by 342 authors with an average document age of 3.11 years. Chinese CAM featured 255 documents by 1857 authors with an average document age of 5.75 years. Iranian CAM showed a negative annual growth rate of -18.05%, while Chinese CAM had a positive rate of 5.65%. The included studies for Chinese CAM span from 2004 to 2024, while those for Iranian CAM range from 2015 to 2024. The most cited Iranian CAM document had 66 citations, and in Chinese CAM, “TONG XL, 2012” and “LIU XL, 2019” had the highest total citations. Decentralized research practices were observed in China, while Tehran and Shiraz universities led clinical trials in Iran.

**Conclusion:**

Our study indicates differing research trends in Iranian and Chinese herbal medicine. Iranian CAM research showed a declining trend, with Tehran and Shiraz universities leading clinical trials, while Chinese CAM research displayed a growing landscape with a more decentralized approach and greater historical prominence among authors.

## Introduction

The origins of traditional medicine, including herbal medicine, trace back to the earliest human civilizations [1]. In the late 19th century, advancements in chemistry and pharmaceuticals led to the extraction of pure chemical compounds for therapeutic use [2]. The rise of synthetic pharmaceuticals sparked renewed interest in medicinal plants, but the long-term use of these drugs revealed significant adverse effects, exemplified by the thalidomide disaster, which resulted in approximately 20,000 children being born with congenital abnormalities [3]. Persian medicine, with a history spanning several centuries, is effective in disease prevention and treatment. Its integration with modern medicine can address various health issues, particularly through the use of medicinal plants [4]. Developing this sector aligns with the World Health Organization's recommendation for affordable and safe medicine [5]. Both ITM and Chinese medicine have significantly influenced ancient medical knowledge [6]. Despite their differences, these two systems have influenced each other historically [7].

Medicine in Iran was prominent even before the Islamic era, with a robust system in the Zoroastrian period, which refers to the time when Zoroastrianism was the dominant religion in Iran, roughly from the 6th century BCE to the 7th century CE [8]. The establishment of Jundishapur University in Khuzestan exemplifies the advanced state of medical professions in pre-Islamic Iran [9]. Post-Islam, scientists like Holly Abbas, Avicenna, and Rhazes instead of Ali Ibn Abbas Ahwazi, Abu Ali Sina, and Zakaria Razi further advanced medicine [10]. ITM, influenced by Greek medicine, became widely recognized [11]. It is based on the principles of Arbaeh, a sophisticated adaptation of Greek medicine [12]. In contrast, TCM, founded on a five-element theory, includes practices like herbal medicine, acupuncture, moxibustion, massage (tuina), and mind/body healing [15]. In China, TCM is integrated with Western medicine and is highly regarded [16]. According to the World Health Organization, 80% of the global population uses plant extracts for primary healthcare [17]. A global survey found that 5% to 74.8% of participants use alternative or complementary therapy [19], with another survey reporting 62.5% utilization [20].

In Iran, individuals with diabetes often use ITM, particularly herbal medicines [21]. Common treatments include vitamin supplements, traditional herbal items, mineral supplements, and food therapy [22]. A recent study found that 6.5% of the population uses AYUSH practitioners, while 7.0% use traditional health care practitioners, with a combined prevalence of 13% [23]. TCM is perceived as valuable for chronic and severe illnesses [24]. Survey findings indicate that 76.3% of TCM users seek it for illness prevention, 67.3% for various medical and health disorders, 34.0% for chronic disease management, and 33.0% for symptom relief. Additionally, 26.9% use TCM regularly, and 26.3% for rehabilitation and sleep problems [25]. Iranian and Chinese traditional medicine have deep historical roots and have gained substantial popularity both among the general public and within the healthcare community. With growing interest from people, healthcare professionals, and researchers, the bibliometric landscape of these fields is evolving rapidly. Consequently, we have undertaken a comparative study to examine the bibliometric trajectories of traditional herbal medicine in Iran and China.

## Materials and Methods

In this bibliometric comparative study, Chinese Complementary and Alternative Medicine (CAM) clinical trials were examined in comparison to Iranian or Persian CAM clinical trials. The investigation exclusively focused on research indexed in Web of Science, with no imposed time limitations. The search syntax used for identifying Randomized Controlled Trials (RCTs) in the field of CAM encompassed terms related to clinical trials, such as "clinical trial," "randomized controlled trial," and "intervention study," were combined with a broad range of keywords associated with Chinese CAM, including "Chinese herbal medicine," "traditional Chinese medicine," "TCM," "acupuncture," "acupressure," "moxibustion," "cupping," "reflexology," "tuina," "qigong," "tai chi," "herbal therapy," "herbal treatment," "herbal remedy," "herbal medicine," "botanical medicine," "phytotherapy," "Chinese medicine," "Chinese therapy," "Chinese treatment," "Chinese remedy," and similar phrases. To ensure the exclusion of non-relevant publications, the search query also included criteria to omit certain document types such as "review," "observational," "qualitative," "bibliometric," "perspective," "retrospective," "meta-analysis," and "protocol." Additionally, this search was confined to articles published in the topic of "INTEGRATIVE COMPLEMENTARY MEDICINE." in WOS. A parallel search was conducted for Iranian/Persian CAM clinical trials, using search terms pertinent to clinical trials and Iranian CAM, and the same exclusion criteria. The studies examined in this analysis were restricted to the geographic regions of Iran and China. Chinese CAM query resulted in 255 studies and Iranian query in 71 studies. The dataset comprised information of author names, article titles, source publications, document types, author-provided and additional keywords, abstracts, author affiliations and addresses, references cited within the articles. Furthermore, it offers information about the articles' citation history, count of times they have been cited, both in the Web of Science Core Collection and across all databases. Data was imported to bibliometrix R application for analysis [26]. The Annual Growth Rate (AGR) of number of articles was provided with a percentage that represents the annual growth rate of the number of articles. A positive AGR indicates growth, while a negative AGR indicates a decline.

Three-Field Plot was used to visually represent associations among authors, affiliations, and keywords for Chinese and Persian CAM separately, providing a view of the relationships within these domains for both practices.

Lotka's Law that is a principle in bibliometrics that describes the distribution of productivity among authors was calculated by bibliometrix. This law suggests that a small number of authors write the majority of the documents, while a larger number of authors write fewer documents.

The collaboration network of co-citations was analyzed by constructed network in Bibliometrix where articles served as nodes connected by edges representing co-citations.

## Results

**Figure-1:**
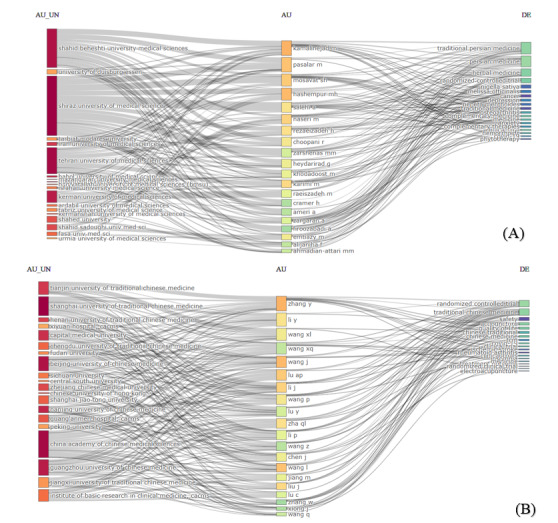
Figure 1. Three-Field Plot of records in (A) ITM and (B) TCM

**Figure-2:**
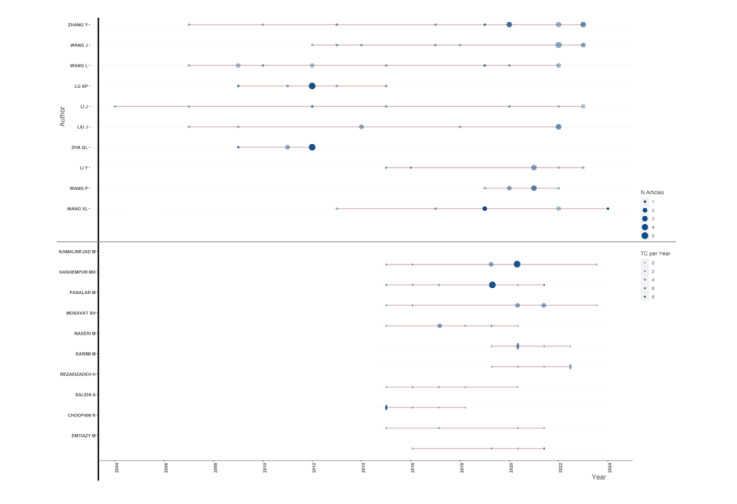
Figure 2. Authors production over time, upper part for China and lower for Iran

**Figure-3:**
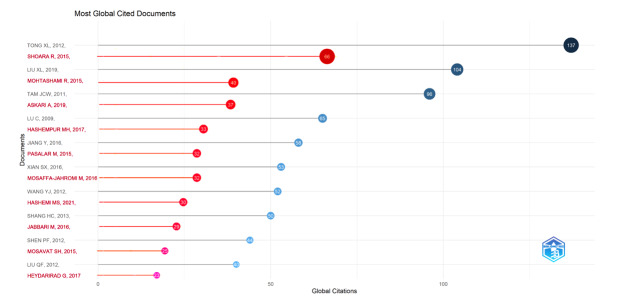
Figure 3. Most global cited papers of TCM (blue colored) and ITM (red colored)

**Figure-4:**
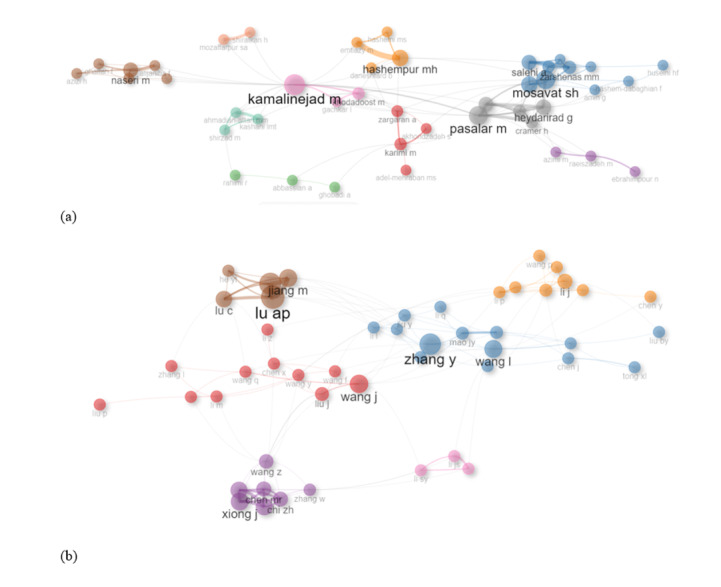
Figure 4. Co-Citation analysis of Chinese and Iranian CAM

**Table:** Table1. Characteristics of Pairs of Records Included in Bibliometric Analysis

**Description**	**Chinese CAM **	**Iranian CAM **
**Timespan**	2004:2024	2015:2024
**Journals**	26	16
**Documents**	255	71
**Annual Growth Rate % **	5.65	-18.05
**Document Average Age **	5.75	3.11
**Average citations per doc **	10.41	9.141
**References**	8657	2931
**Author's Keywords **	682	236
**Authors**	1857	342
**Co-Authors per Doc **	9.85	6.63
**International co-authorships % **	16.86	12.68

For study of Iranian or Chinese herbal medicine, we exported data including affiliation, author information, cited references, corresponding author, DOI, document type, journal, language, number of cited references, publication year, science categories, title, and total citations, with no missing data in any of them. But there was 1.41% missing data and 2.82% missing data for Keywords for abstracts of Iranian studies; while there was 32.16% missing data in keywords of Chinese medicine. Over the timespan from 2015 to 2024, a total of 342 authors contributed to 71 documents published in 16 different sources for Iranian CAM. There were 2931 references cited across these documents. International co-authorship was at 12.68%. The average document age was relatively low at 3.11 years, suggesting a focus on recent research. However, the annual growth rate is negative at -18.05%, which may indicate a decline in publications over the years. On average, each document had 6.63 co-authors and received approximately 9.141 citations.

For Chinese CAM, from 2004 to 2024, with 1857 authors contributing to 255 documents, featuring 682 keywords, 26 sources, 0 single-authored documents, 8657 references, and an international co-authorship rate of 16.86%, with an average document age of 5.75 years, an annual growth rate of 5.65%, 9.85 co-authors per document, and an average of 10.41 citations per document. As shown in Figure-1 CAM research practices in form of clinical trials are decentralized in China and many institutes are working on the issue; but in Iran, Tehran and Shiraz universities have most share of the clinical trials on the CAM. The majority of authors (approximately 79.5%) have written only 1 document. A smaller proportion of authors (about 12.6%) have written 2 documents. Figure-2 shows the list of authors with repeated publications through the timespan and shows that in comparison to Iranian authors, Chinese authors tend to have a longer history and greater prominence in the field of clinical trials in complementary medicine. The first ranked Iranian CAM document, authored by SHOARA R and published in 2015 [27], has a total of 66 citations, an average of 7.33 citations per year, and a normalized TC of 2.10. The second document, authored by MOHTASHAMI R and published in 2015 [28], has 40 total citations, an average of 4.44 citations per year, and a normalized TC of 1.27. In Chinese CAM, the data reveals that "TONG XL, 2012" [29] and "LIU XL, 2019" [30] papers have the highest total citations, with 137 and 104, respectively, while "LIU XL, 2019" has the highest normalized total citations at 5.98. Most cited articles in both practicing manners are shown in Figure-3. Collaboration network of co-citations in Chinese CAM studies was clustered in 7 clusters and Persian ones were in 12 clusters, as shown in Figure-4. There were no interactions in co-Citation analysis of Chinese and Iranian CAM.

## Discussion

Our study indicated differing research trends in Iranian and Chinese herbal medicine. Iranian CAM research showed a declining trend, while Chinese CAM research displayed a growing landscape with a more decentralized approach and greater historical prominence among authors. The scientific publication of articles in medical sciences is a vital indicator of a country's research output and academic excellence [31][32]. A comparative analysis of Iran and China's publication trends reveals distinct patterns, with China dominating the global landscape in terms of sheer volume. According to a systematic review, Chinese authors accounted for 73.75% of the corresponding authors in non-Cochrane retracted systematic reviews. In contrast, Iran's publication output, although significant, lags behind China's, with a notable presence in fields like traditional Persian medicine and cautery [31][32].

This approves what we find on our research. A study on the comparison of kaiy in Persian medicine and moxibustion in Chinese medicine highlights the similarities and differences between these traditional practices [32]. In terms of specific medical fields, a bibliometric study on neurosurgical publications in high-impact medical journals reveals a growing trend of publications from China, while Iran's output is relatively limited [33].

The historical exchange of Chinese herbal drugs with the Islamic world, as discussed in another study, underscores the significance of traditional medicine in shaping global healthcare practices [34]. The history of Traditional Chinese Medicine (TCM) and Iranian alternative medicine has taken different paths. In the past, there was a significant exchange of medical knowledge and trade between China and the Islamic world, especially during the "Golden Age of Islam" (8th-13th century). Famous Muslim doctors like Avicenna and Rhazes praised Chinese herbal remedies, which were used in similar ways in both Chinese and Islamic medicine. In fact, Avicenna's book "Canon of Medicine" mentions Chinese herbal imports, such as cinnamon and ginger, 46 times [34].

The field of bibliometrics has made significant contributions to our knowledge of scientific fields and the development of policy [35]. Our study conducted a comprehensive analysis of Iranian and Chinese herbal medicine research. Data was mostly complete, with the exception of missing keywords. In the period from 2015 to 2024, Iranian CAM research appeared to focus on recent developments but showed a negative annual growth rate. In contrast, Chinese CAM research demonstrated growth, marked by collaboration and an emphasis on clinical trials. According to the results of bibliometrics study of Huang et al. (2015) in TCM, 4364 articles were published by a single journal Zhongguo Zhong Yao Za Zhi [36]; while Iranian.

The results of Musa et al. (2022) studies showed that after 1990, there was a yearly rise in studies conducted on traditional herbal therapy, which was accompanied by a commensurate increase in global citations. In total, 22,071 writers contributed to all the publications. China, Japan, and India emerged as the leading countries of origin for research on traditional herbal medicine. Notably, Beijing University of Chinese Medicine, China Academy of Chinese Medical Sciences, and China Medical University were identified as the top affiliations.

The National Natural Science Foundation of China, the National Key Research and Development Program of China, the Ministry of Science and Technology of the People's Republic of China, and the Ministry of Science and Technology, Taiwan were the primary financing agencies, each having over 100 documents [37]. Zhong et al.'s (2023) findings showed that research on TCM and nanoparticles is increasing with an increasing number of publications over the years. This type of research is mainly conducted in China and international cooperation is limited. Most research in this field focuses on single herbal compounds, while studies on nanoparticle formulations of traditional herbal versions are relatively scarce [38].

As Consentino et al. (2018) noted, a total of 122 articles were published in 2007, the peak year, and the data showed an annual growth rate of almost 33%. China dominated the distribution of scientific output (76.1%), followed by the USA (3%), and South Korea (2.1%). More than half of the citations came from Chinese publications, yet the impact factor of these journals was very low. Phytotherapy (55%) and acupuncture (40%) were the most often mentioned treatments in the articles' keyword sections [39].

The number of published articles in Persian Medicine had significant growth in the last years. Moeini et al. (2015) study showed that 502 Articles were found up to the end of 2015; 54.3% original articles, 26% review, 13.7% letter to editor, 29% biographical and historical articles. Pharmacological and phytochemical studies were the most published articles (14 .7%). Between 139 journals which published these articles, 59% have Impact factor (IF) and highest IF belonged to the Lancet neurol J (23 .46). More than one citation was reported in 43.7% of articles and the most citation belong to the one of the published articles in year 2012 (23 citation). H -Index of all collected articles was 12 [40].

According to Saberi et al.'s (2023) research, the upward trend in journal publication and citations is followed by a declining tendency. As a result, there will be a larger volume of articles in the magazine between 2010 and 2016, followed by a decline. Most of the top authors are from Tehran and Isfahan Universities of Medical Sciences. There are 74 articles on traditional medicine, 46 articles on traditional Iranian medicine, and 45 articles on traditional Persian medicine [41]. This shows that sources where publication are retrieved like PubMed, WOS, and Science direct and even the timeline change the bibliometric results extensively.

## Conclusion

In conclusion, our comparative analysis of Iranian and Chinese herbal medicine research reveals notable differences in research trends. These differences can inform policymakers, researchers, and practitioners about the strengths and areas for improvement in each country's CAM research landscape. The study acknowledges the deep historical roots of traditional medicine in both China and Iran. Findings show how each country's traditional medicine clinic trials are evolving and contributing to the global CAM landscape.

## Conflict of Interest

None.
